# Diet and Chronic Non-Cancer Pain: The State of the Art and Future Directions

**DOI:** 10.3390/jcm10215203

**Published:** 2021-11-08

**Authors:** Katherine Brain, Tracy L. Burrows, Laura Bruggink, Anneleen Malfliet, Chris Hayes, Fiona J. Hodson, Clare E. Collins

**Affiliations:** 1 School of Health Science, College of Health, Medicine and Wellbeing, University of Newcastle, Callaghan, NSW 2308, Australia; katherine.brain@newcastle.edu.au (K.B.); tracy.burrows@newcastle.edu.au (T.L.B.); 2 Priority Research Centre for Physical Activity and Nutrition, University of Newcastle, Callaghan, NSW 2308, Australia; 3 Hunter Integrated Pain Service, Newcastle, NSW 2300, Australia; laura.bruggink@health.nsw.gov.au (L.B.); chris.hayes@health.nsw.gov.au (C.H.); fiona.hodson@health.nsw.gov.au (F.J.H.); 4 Department of Physiotherapy, Human Physiology and Anatomy, Faculty of Physical Education & Physiotherapy, Vrije Universiteit Brussel, 1050 Brussels, Belgium; anneleen.malfliet@vub.be; 5 Pain in Motion International Research Group, 1000 Brussels, Belgium; 6 Research Foundation Flanders (FWO), 1000 Brussels, Belgium; 7 Department of Physical Medicine and Physiotherapy, University Hospital Brussels, 1090 Brussels, Belgium

**Keywords:** nutrition, diet quality, chronic non-cancer pain

## Abstract

Nutrition plays an important role in pain management. Healthy eating patterns are associated with reduced systemic inflammation, as well as lower risk and severity of chronic non-cancer pain and associated comorbidities. The role of nutrition in chronic non-cancer pain management is an emerging field with increasing interest from clinicians and patients. Evidence from a number of recent systematic reviews shows that optimising diet quality and incorporating foods containing anti-inflammatory nutrients such as fruits, vegetables, long chain and monounsaturated fats, antioxidants, and fibre leads to reduction in pain severity and interference. This review describes the current state of the art and highlights why nutrition is critical within a person-centred approach to pain management. Recommendations are made to guide clinicians and highlight areas for future research.

## 1. Introduction

Chronic non-cancer pain (CNCP) is defined as pain that persists for more than three months, which exceeds the time it typically takes for tissues to heal [1]. Globally, the prevalence of CNCP is approximately 20%, with a higher prevalence among vulnerable populations such as the elderly and those from culturally and linguistically diverse backgrounds (≥40%) [2,3,4]. In 2010, the economic burden of CNCP in the United States was reported to be $635 billion, exceeding that of heart disease ($309 billion), diabetes ($188 billion), and cancer ($243 billion) [5]. In Australia, the cost of chronic pain in 2018 was $139.3 billion and expected to increase to $215.6 billion by 2050 [4]. CNCP is a major burden on both individuals and the community due to absenteeism and loss of productivity [4]. In Australia in 2018, $48.3 billion of the financial cost associated with CNCP was attributed to productivity losses and $66.1 billion was attributed to reductions in quality of life [4]. CNCP also causes increased stress on the health care system, as many people experiencing pain have exacerbations of other chronic health conditions requiring specialised treatment. In 2019, self-reported data from 72 adult CNCP services (30,000 patients) across Australia and New Zealand reported approximately 40% of patients had mental health issues, 23% had digestive diseases, 22% had high blood pressure and/or high cholesterol, and 10% had diabetes [6]. Individuals who live with pain can find it difficult to move about and socialise. Pain also impacts their mood, ability to shop and cook, and the food and drinks they consume.

A whole-person approach to pain management is a patient-centred framework that encourages the adoption of active strategies to address biopsychosocial and lifestyle modulators of pain experiences [7]. In this broad context, there is recognition of the multidirectional relationships between diet, mental health, sleep, food preparation, and mobility [7]. Addressing these dimensions of pain experience in specialist multidisciplinary pain services reduces pain and improves quality of life [6,8]. There are, however, challenges in implementing multidisciplinary approaches in primary care [9]. Nutrition is a central component of the whole-person approach and emerging evidence, explored in this review, suggests that dietary interventions can be effective in improving quality of life and managing CNCP, as well as comorbid mental and physical health problems [10].

This state-of-the-art overview explores the role of diet in CNCP. The available evidence is reviewed with the aim of helping clinicians translate findings into practice and assisting researchers to optimise the design of future trials and implementation studies.

### 1.1. Diet, Pain, and Systemic Inflammation

Persisting low-grade systemic inflammation is associated with CNCP and multiple comorbid chronic health conditions. Diet plays a complex role in modulating systemic inflammation. Knowledge is expanding rapidly in this area and multiple links between diet and inflammation have been identified. Metabolic mechanisms associated with post prandial hyperglycaemia and frequent and prolonged rises in plasma insulin levels, influenced by dietary intake, can produce systemic inflammation [11,12]. This has been shown in insulin-resistant states where increasing adiposity is associated with the increased secretion of pro-inflammatory cytokines in adipose tissue, liver, and skeletal muscle [13].

There are several mechanisms associated with fat metabolism. An excess of omega-6 fatty acids relative to omega-3 fatty acids loads the arachidonic acid pathway and contributes to a pro-inflammatory state [14,15]. The body requires both omega-3 and omega-6 fatty acids, ideally in a ratio of approximately 1:1 [16]. Dominance of omega-6 polyunsaturated fats in Westernised diets over the last few decades has led to ratios of omega-3 to omega-6 in the range of 1 to 15–30, which has been shown to promote systemic inflammation [14]. Industrial trans fats, or hydrogenated oils, also promote inflammation and raise LDL cholesterol and lower HDL cholesterol [17,18].

In the context of CNCP, an aspect of systemic inflammation of particular interest manifests in the central nervous system. This neuroinflammation is mediated by neuroglia cells, which are found in the brain and central nervous system [19]. They are thought to be activated by overall poor dietary patterns (i.e., energy-dense, nutrient-poor diets) through a variety of mechanisms such as oxidative stress, peripheral inflammation, and changes in the gut microbiome [19]. This leads to central nervous system sensitisation, a dominant contributor to CNCP [19,20]. The corollary of this is that the adoption of a high-quality diet facilitates normalisation of glial activity and reduced central nervous system inflammation and sensitisation [19].

Alterations in the gut microbiome and associated auto-immune mechanisms also influence systemic inflammation. A range of mechanistic, animal, and observational human studies have found that changes in gut microbiota can influence immune function and may contribute to an increased risk or severity of auto-immune diseases [21]. Increased intestinal permeability potentially allows the translocation of bacterial fragments such as lipopolysaccharides, which can trigger inflammation and auto-immune responses [21].

While metabolic pathways can produce inflammation, they can also have anti-inflammatory activity and reduce oxidative stress [19]. Non-nutritive bioactive compounds such as polyphenols mitigate oxidative stress and inflammation, as well as modulating pain experiences [22]. One such mechanism operates through the inhibition of COX-2 in neuromodulating pathways [22]. Polyphenols are found in a range of foods such as fruits, vegetables, whole grains, cocoa, tea, coffee, and alcohol [23]. Food’s rich in polyphenols, such as cherries, strawberries, blueberries, and plums, have been used in a variety of clinical studies showing anti-inflammatory benefits, as well as cardio-metabolic benefits and neuroprotective effects [24,25,26,27,28]. Dietary fibre intake and the consequent colonic production of short chain fatty acids also reduces inflammation through its beneficial role in the gut microbiome–brain axis and in immunomodulation [22].

As such, dietary factors mediate systemic inflammation and so therapeutic focus should be placed on reducing inflammation through optimising overall dietary quality, addressing the ratio of omega-3 to omega-6 PUFAs, and increasing the intake of polyphenols and fibre.

### 1.2. Diet, Pain, and Comorbidities

Systemic inflammation is linked with CNCP and multiple other comorbidities impacting both physical and mental health [6,29,30,31]. These varied conditions include type 2 diabetes, cardiovascular disease (CVD), respiratory and kidney disease, obesity, cancer, non-alcoholic fatty liver disease, autoimmune disorders, neurodegenerative disorders, and depression [32,33,34]. The specific mechanisms and predominant sites of inflammation, along with the genetic and epigenetic vulnerabilities of the person, influence disease expression. For example, oxidative stress may exacerbate neuropathy [35]. Changes in the vascular endothelium are correlated with cardiovascular disease and metabolic syndrome [36,37]. Neuroinflammation involving immune cells such as glia and cytokine cascades [38,39,40] plays a role in the central sensitisation that is strongly correlated with CNCP.

Systemic inflammation can contribute to CNCP at multiple levels impacting both structural tissues and the nervous system. In osteoarthritis, for example, inflammation is expressed within the affected joint [41] in addition to neuroinflammation in the central nervous system [42].

In a clinical audit in 2017 at a tertiary pain service in Australia, 64% of patients reported having two or more comorbidities [29]. This is supported by a Scottish primary care study which found that 46% of patients presenting with CNCP had three or more long-term conditions [43]. A cross sectional study conducted on a sample of 3000 individuals in Germany also found that those suffering from depression were three times more likely to experience non-neuropathic chronic pain (18%) and six times more likely to experience neuropathic pain (7%) [44]. People with obesity, diabetes, hypertension, and cerebrovascular disease were also at a higher risk of having non-neuropathic chronic pain [44]. A recent systematic review of 20 studies found that people experiencing chronic musculoskeletal pain were almost twice as likely to report having CVD than those people without chronic musculoskeletal pain [45]. Another meta-analysis found that people with diabetes were 1.4 times more likely to report lower back pain and 1.2 times more likely to report neck pain compared to those without diabetes [46]. There is also an association between CNCP and obesity. This is evidenced in two large studies conducted in the United States of America in which it was found that those with a higher Body Mass Index (BMI) were more likely to self-report moderate and severe pain intensity [47,48]. Participants who were classified as obese (Body Mass Index ≥30 kg/m^2^) were approximately 1.3 to 2 times more likely to experience pain [47,48]. Obesity can contribute to pain via increased mechanical load in addition to pro-inflammatory mechanisms [49]. Pain can contribute to obesity by interfering with food preparation and healthy dietary choices.

Given the prevalence of nutrition-related comorbidities associated with CNCP and the overlap of the underlying mechanisms, it is important to consider the role of nutrition in simultaneously reducing the severity and risk of CNCP and other chronic health conditions. Many of these conditions and their associated risk factors can be modulated through changes in diet.

### 1.3. Dietary Intake of People Experiencing Pain

A limited amount of research has assessed dietary intake in people experiencing CNCP. The studies that do exist largely report on diet quality, total energy intake, and macronutrient distribution. A study by VanDenKerkhof et al. analysed data from the British Birth Cohort Study (*n* = 89,673, aged ≥45 years, 12% with CNCP) and found that fruit and vegetable consumption of women experiencing CNCP pain was more likely to decrease over time, compared to women with no pain [50]. Overall diet quality was lower in women with CNCP (≤1 serve/week of fruit and vegetables and ≥1 serve/day of fatty foods and chips), compared to women without pain [50]. A study conducted by Collins et al. examined diet-related survey data from 10,000 Australian women aged 50–55 years [51]. Findings showed that poorer diet quality was associated with higher pain scores as reported using the pain subscale within SF-36 [51]. Conversely, higher diet quality was associated with lower pain levels [51]. Long term opioid use is associated with excessive energy intakes as shown in a study conducted by Meleger et al., where one third of male and half of female patients receiving long-term opioid therapy were exceeding recommended energy intake targets [52]. A pilot study conducted in 2019 found that at baseline, participants’ mean percentage of energy derived from core foods (fruit, vegetables, breads, and cereals, meat and meat alternatives, and dairy and dairy alternatives) was 58% and their mean percentage of energy from energy-dense nutrient-poor foods (e.g., confectionary, sugar sweetened beverages, and takeaway foods) was 42% [53]. Ideally, at least 85–90% energy should come from nutrient-rich core foods and no more than 10–15% from energy-dense nutrient-poor foods [54,55]. The intervention in this pilot study consisted of 6 weeks of personalised dietary consultations and cherry juice high in antioxidants vs. a placebo fruit (apple) juice [53]. After 6 weeks, all groups had a statistically significant increase in percentage of energy from core foods (63%) and a reduction in percentage of energy from energy-dense, nutrient-poor foods (37%) [53]. The group that received the personalised dietary consultations had a significant reduction in percentage of energy from total fat (−3.36%) compared to the control group (+2%) [53]. Participants who received the cherry juice did no better than those who received the apple juice [53].

### 1.4. Diet and the Whole-Person Approach to Pain Management

**Figure 1:**
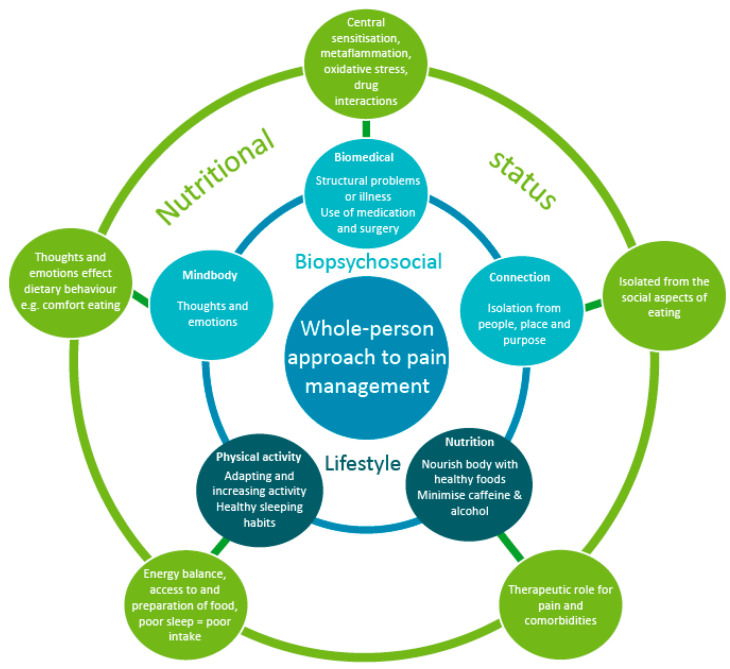
The relationship between nutrition and the whole-person approach to pain management (adapted and reprinted with permission).

The biopsychosocial and lifestyle factors that influence pain all interact, and these factors rarely stand alone in terms of contribution to pain experiences. Figure 1 depicts the relationship between nutrition and the whole-person approach to pain management.

#### 1.4.1. Diet and Biomedical Aspects

There is a complex relationship between the biomedical and psychosocial aspects of pain and nutrition. From a biomedical perspective, as previously discussed, dietary intake can affect pain by modulating systemic inflammation and oxidative stress, as well as by its impact comorbid conditions.

The adverse effects of medications used for pain and other chronic health conditions can be substantial and add to nutritional challenges. Opioid medications commonly reduce motility, delay transit and gastric emptying, and suppress androgen and adrenal function [56]. This in turn can adversely impact metabolism and increase feelings of fullness, bloating, nausea, and constipation. Mechanism-based studies conducted in animals and humans have shown that non-steroidal anti-inflammatory drugs (NSAIDs) can increase gut permeability, inflammation, and the risk of gastrointestinal injury (e.g., ulcers) [57,58]. Antidepressants and anticonvulsants commonly used for pain management are also associated with gastrointestinal side effects such as nausea, constipation, diarrhoea, and changes in appetite [59,60]. Medications can also impact the gut microbiome. Antibiotics and proton pump inhibitors, for example, can have major adverse impacts on microbiome diversity [58].

Tapering and ceasing, or minimising the dose of pain related medications, will improve gastrointestinal and nutrition-related problems [61]. Adequate intake of soluble and insoluble fibre and water can assist in relieving the side effects of constipating medications [62]. More information about fibre and fluid can be found in Section 2.2.5 and Appendix A.

#### 1.4.2. Relationships between Diet, Mental Health, and Lifestyle

Mental health comorbidities such as anxiety and depression, as well as feelings of isolation and loss of connection to people, place, and purpose, are also common in people experiencing CNCP [6,63,64]. Self-reported data from 72 adult pain services (30,000 patients) in Australia and New Zealand shows that 40% of patients have depression, anxiety, and/or post-traumatic stress disorder [6]. A bivariate adjusted analysis of the Canadian Longitudinal Study of Ageing (*n* = 28,000) found that those who were socially isolated and/or lonely had an increased likelihood of psychological distress relative to those who were neither isolated nor lonely [65]. Subsequent studies have found that interventions targeting social isolation have led to significant improvements in self-reported pain intensity and emotional and physical functioning [63,64].

Mental health issues and isolation can lead to changes in dietary behaviours such as comfort eating, low motivation for meal preparation, loss of appetite, and lack of meaning around meal times. Qualitative data show that people experiencing CNCP report using emotional eating or binge eating behaviours as a response to their pain [66]. Participants reported that this often coincides with depression and guilt [66]. Depression and anxiety are associated with overall low diet quality [67,68]. Low diet quality is associated with lower intakes of key essential macro and micronutrients often found in foods such as fruits and vegetables [51,69].

Overeating is also associated with CNCP. Mechanisms for overeating in response to pain are likely highly varied due to the clustering of a range of comorbidities in this population group, which may include depression and anxiety. Overconsumption could be related to hedonic hunger triggered by physical pain, as well as emotional eating as a coping strategy [66]. Consuming food may elevate low mood or provide a distraction from anxious or traumatic thoughts via activation of brain reward pathways involving neurotransmitters such as dopamine [70]. Data from a survey of over 200 adults with CNCP reported that approximately 12% of respondents ate more to feel better when they experienced pain [70]. In another study of 126 veterans, the Yale Emotional Overeating Questionnaire (YEOQ) was used to examine overeating responses to physical pain [71]. Approximately 43% of participants had engaged in at least one overeating episode in response to pain in the past month and 14% engaged in this behaviour daily [71]. This study proposed that those with higher pain interference are more likely to have depression and may have maladaptive pain-related coping, including overeating [71]. This may be due in part to associations between higher pain catastrophizing, low distress tolerance, and higher levels of unhealthy eating [72].

Pain can lead to reduced mobility and functional strength, which in turn can make shopping, cooking, and preparing meals difficult and may exacerbate pain [73]. Given the range of living conditions of those with CNCP, there may be increased vulnerability of some population groups to these factors such as those living alone, in group homes, or in aged care. Decreased mobility due to pain often means regular employment is difficult, and there may be large periods of unemployment contributing to financial burden. Reliance on takeaway or convenience foods may be an appealing solution to some people experiencing pain. However, this can lead to low diet quality.

Pain can also significantly impact sleep. This may include quantity, quality, sleep hygiene, and how long it takes to get to sleep, which are all important elements that need to be considered. A lack of restorative sleep leads to increased tiredness, caffeine consumption, overall daily energy intake, and fat, protein, and carbohydrate intake, and can also lead to impaired hormone regulation [74,75]. For example, leptin may be reduced, with a consequent decrease in satiety signals to the brain. In addition, levels of ghrelin, a ‘hunger hormone’, may be increased by lack of sleep [74,76]. Poor sleep can also affect glucose tolerance and insulin levels, with an increased risk of type 2 diabetes, which is highly prevalent in people experiencing CNCP [6,46,74,76].

It is clear that nutrition does not stand alone in the management of pain, but there is equally a need to recognise that food has important direct and indirect influences on the whole-person pain management approach.

#### 1.4.3. Diet and the Whole-Person Approach to Pain Management and Behaviour Change

It is important to consider tips and strategies to address dietary behaviours as well as dietary intake. Given the complexities surrounding the relationship between nutrition, pain, and the whole-person approach to pain management, behaviour change strategies are well placed to support people to change their habits. The Behaviour Change Model is an evidence-based approach that incorporates the overarching aspects of environment, policy, and regulation, combined with clinician-delivered interventions, and patient factors of capability, opportunity, and motivation [77]. At an individual patient level, it is vital that health professionals identify their patients’ capabilities, opportunities, and motivations to help set specific nutrition goals and facilitate successful behaviour change [77]. Health professionals can use the sources of behaviours as a way to identify patient’ barriers and/or facilitators. They can then assist patients to overcome barriers or harness facilitators to ensure successful behaviour change [77]. For example, your patient may not know the relationship between diet and CNCP (capability), they may not have time or access (opportunity), and they may not have the belief or confidence to change (motivation).

To ensure consistent and high-quality care, it is important to follow a process to comprehensively assess dietary intake, take challenges into consideration, implement strategies, and monitor progress. The first step of this process is to assess dietary intake. There are a variety of tools including brief dietary screeners, such as the Healthy Eating Quiz [78], that provide an indication of overall diet quality, and more comprehensive tools (e.g., food frequency questionnaires, food records, or 24-h recalls), that can be used to assess the adequacy of food and nutrient intakes relative to national recommendations. Which one should be used depends on the situation and purpose? Many variations on these tools are available to the public online or via apps, which makes it easier for patients to access them. Some online tools and apps can also provide instant analysis. The next step is comparing the dietary assessment to recommendations such as national guidelines or nutrient reference values. Comparison to recommendations allows the identification of areas for improvement and these are often the basis of goals. Exploring barriers to and motivators for change will assist making a SMART (Specific, Measurable, Achievable, Relevant, and Time-bound) goal that is realistic and achievable. There are a number of potential barriers that need to be taken into consideration such as socioeconomic and cultural preferences, food availability, mental health and mobility issues, and poor health literacy [79]. These barriers need to be addressed with appropriate and relevant strategies. Working with patients to identify relevant barriers and strategies will make it easier for patients to achieve their goals. This can be done using the COM-B model. For example, identifying culturally and linguistically diverse (CALD) services in your area can help assist CALD patients. Self-monitoring progress is helpful for patients to maintain their level of awareness and motivation towards change. Self-monitoring also gives clinicians an indication on how their patient is progressing and allows the revision of goals if needed.

## 2. State of the Art

### 2.1. Nutrition Interventions for People Experiencing Chronic Non-Cancer Pain

Research from a pilot study (evidence level 1c) (Table 1) conducted in 2019 found that a personalised dietary intervention that included a dietary assessment, dietary advice for pain management, and strategies to overcome barriers to assist with behaviour change that was delivered by a dietitian had a clinically meaningful effect on self-reported pain interference and pain self-efficacy [53]. Participants also had improvements in quality of life and dietary intake [53]. However, given that this was a pilot study, the intervention needs to be implemented and tested in fully powered trials. Another quasi-experimental study (evidence level 2d) in a cohort of people with chronic musculoskeletal pain found that an 8-week plant-based diet led to a statistically significant reduction in pain (mean change 3.14, *p* = 0.0001) measured on a numerical pain rating scale [80], although this study had a small sample size (*n* = 14).

A recent systematic review (evidence level 1b) collated and summarised experimental studies exploring the effect of dietary interventions on chronic non-cancer musculoskeletal pain, arthritis, and fibromyalgia [82]. Through a synthesis of results from 43 studies overall, a positive effect was found for a number of whole food dietary interventions (i.e., foods commonly found in the diet, excluding nutraceuticals) with an average reduction in pain score, −0.44, *p* < 0.0001 [82]. Other systematic reviews in people with chronic musculoskeletal pain, arthritis, and fibromyalgia have found similar results. Elma et al. found that in 12 experimental and observational studies, vegetarian, vegan, weight loss, or peptide diets were associated with improved pain outcomes (evidence level 1b) [83]. Two other systematic reviews (evidence level 1b) in people with arthritis (*n* = 7 studies) and fibromyalgia (*n* = 7 studies) included studies with interventions focused on diets that are predominantly plant rich and/or contain anti-inflammatory aspects (e.g., Mediterranean diet, omega-3, or antioxidants) where participants had a reduction in pain outcomes [84,86]. Commonalities among all of these interventions include a focus on improving diet quality and nutrient density. This is supported by another systematic review of 71 studies (evidence level 1b) [85], which found that studies that used a dietary intervention to alter overall intake, particularly vegetarian or Mediterranean diets, or the quality of a specific nutrient such as fat or protein, achieved statistically significant reductions in pain intensity [85].

Three other reviews, collectively including 218 studies (evidence level 2b) have also explored the role of nutrition in CNCP. However, these studies include a large number of mechanism-based studies, and have summarised the literature, rather than provided a synthesis of results [35,87,88]. When comparing the summaries provided in these reviews to the results from the systematic reviews outlined above, it is still evident that the literature points towards optimising diet quality, increasing consumption of core foods such as fruit, vegetables, breads and cereals, meat, dairy, and their alternatives and reducing energy-dense nutrient-poor foods such as confectionary, sugar sweetened beverages, and processed meats.

Among the systematic reviews conducted in this area, many share limitations, with substantial heterogeneity among pain “conditions” and dietary interventions. Intervention studies that include participants with multiple types of CNCP are rare and it is more common to find studies which explore the impact of nutrition on sub-types such as arthritis, musculoskeletal pain, fibromyalgia, or gastrointestinal pain (e.g., inflammatory bowel disease (IBD) and irritable bowel syndrome (IBS)). There is a challenge in balancing nutritional recommendations relevant to the breadth of people with CNCP with a focus on more specific recommendations for particular diseases or individuals. The majority of the studies included in these systematic reviews were also of low methodological quality and used unidimensional tools to measure pain outcomes. This indicates the need for more and higher quality studies that use multidimensional tools to measure pain outcomes to ensure all aspects of pain are considered.

Given that this is an emerging field of research, there are also a number of expert consensus papers (evidence level 5b) on this topic that should be considered. A common aspect of all of these papers (*n* = 4) is the focus on systemic inflammation [19,22,79,89]. Consequently, these papers suggest, consistent with healthy eating principles for chronic disease reduction, that dietary intake should include fruits and vegetables, food rich in antioxidant nutrients (in particular polyphenols), olive oil, nuts, legumes, and adequate intake of micronutrients (omega-3, vitamin B12, vitamin D and magnesium) [19,22,79,89]. These papers also acknowledge the challenges people with CNCP face in achieving healthy dietary patterns and behaviours. There is a need to consider socioeconomic and cultural differences, food availability and psychological or physical difficulties.

Nutrition interventions are highly variable in clinical settings. The availability of dietitians is often a significant limiting factor. Other allied health professionals have variable nutrition training and consumers are often left to seek dietary advice on their own. The following section will provide appropriate evidence-based recommendations for a range of health professionals.

### 2.2. Recommendations for Clinicians

#### 2.2.1. Dietary Assessment

Dietary assessment is extremely important, as acknowledged in Philpot et al. (evidence level 5b). Pain services would benefit from working with dietitians to access their skills in dietary assessment [79]. Dietary screeners which assess diet quality (e.g., The Healthy Eating Quiz) [78] along with an assessment of psychological, physical and medical issues allows clinicians to look at the relationship between diet and pain experiences and diet-related risk factors with other chronic diseases. This also allows clinicians to identify some of the socioeconomic, physical, and psychological barriers to healthy eating that are common in people experiencing CNCP [22,79].

A common theme arising in the evidence is the potential role of vitamin and mineral deficiencies, such as Vitamin D, Vitamin B12, and magnesium, in pain experiences [22,79,88]. The only non-invasive way to determine if a patient has a micronutrient deficiency is through systematic dietary assessment that reflects usual dietary intake conducted by a dietitian. Some practical tips on dietary assessment and identification of micronutrient deficiencies are available in Appendix A.

#### 2.2.2. Optimise Diet Quality

All the systematic reviews exploring the role of nutrition in pain management (evidence level 1b) emphasised optimising diet quality [82,83,84,85,86]. Poor diet quality is associated with high consumptions of energy-dense nutrient foods that lack key nutrients found in core foods such as fruits, vegetables, breads and cereals, meat, dairy, and their alternatives. Globally, poor dietary intake is the one of the top modifiable risk factors for morbidity and mortality [90]. Specifically, high sodium intake and low intake of whole grains, fruit, nuts, and seeds are the top three leading risk factors [90]. In line with the evidence presented in this paper, these foods contain fibre, vitamins, and antioxidants that are associated with reducing pain experiences [89]. Given that over 90% of Australians and Americans do not follow their respective country’s evidence-based dietary guidelines [91,92], the first step to improving diet quality is to increase adherence to national dietary guidelines. While national dietary guidelines are not specific to CNCP management, they promote healthy eating and lifestyle behaviours which may better translate for those experiencing CNCP.

#### 2.2.3. Consume Fruit and Vegetables Rich in Phytonutrients to Reduce Oxidative Stress

All of the systematic reviews (level 1b) included a large number of studies that used plant-rich eating (e.g., vegetarian or vegan dietary patterns), anti-inflammatory, and Mediterranean diets [82,83,84,85,86]. A major component of all of these dietary patterns are fruits, vegetables, and whole grains, which contain phytonutrients with antioxidant properties. To maximise consumption of phytonutrients and polyphenols it is important to consume a wide range of different coloured fruits and vegetables [89]. However, as acknowledged in some of the expert review evidence (level 5b), there are additional considerations that may impact someone’s ability to include a wide range of fresh and colourful fruit and vegetables in their diet [22,79,89]. This can include potential exacerbation of pain through preparation and cooking, and/or lack of motivation to shop and cook [89]. Practical tips to address this are found in Appendix A.

#### 2.2.4. Consume Long Chain and Monounsaturated Fats (e.g., Omega-3 and Olive Oil)

A number of experimental studies included in the systematic reviews (level 1b) that have been synthesised for this paper have shown that long chain and monounsaturated fats, especially omega-3 fats and olive oil reduce pain [82,83,84,85,86]. Suggestions on how to increase omega-3 fats and olive oil can be found in Appendix A.

#### 2.2.5. Increase Fibre and Water Intake

Fibre is essential for proper digestion and maintenance of a healthy microbiome. Fibre and fluid work together to promote bowel health. It is important that when your patient increases their fibre intake, they also increase their fluid intake. Fibre is found in fruits, vegetables, and whole grains, which are the main components of the plant rich dietary interventions included in the systematic reviews that make up the evidence for this paper.

#### 2.2.6. Reduce and Limit Ultra-Processed Food and Added Sugar Intake

Ultra-processed and sugar-dense foods and drinks contain very high amounts of energy and negligible amounts of beneficial nutrients. These foods are often high in fat, salt, and sugar, and in the case of beverages, caffeine, which can impact sleep. Some examples include soft drinks, sweet or savoury packaged snacks, confectionary, and reconstituted meat products. These foods are often high in fat, salt, and sugar, and in the case of beverages, caffeine. These nutrients can have a number of effects including increasing circulating inflammatory markers and oxidation [11,93] and impacting sleep. In relation to sugar consumption, the World Health Organisation (WHO) recommends that adults limit intake of ‘free sugar’ including table sugar, honey, syrups, and sugar-sweetened beverages to less than 10% of total energy [94].

#### 2.2.7. Other Nutritional Considerations

As shown in Figure 1, nutrition also encompasses other dietary factors such as caffeine and alcohol. Caffeine is commonly consumed in tea and coffee, and evidence shows that low to moderate consumption of coffee is associated with reduced mortality [95]. Tea and coffee contain other phytonutrients such as polyphenols, and it may be that these are responsible for their health benefits [95]. Coffee consumption later in the day or in high doses (>200 mg/serve or >400 mg/day) may increase anxiety and reduce quality of sleep, both of which can negatively influence pain experiences [95]. Decaffeinated options are a good alternative to avoid increased anxiety or sleep issues. Other sources of caffeine or guarana such as soft drinks and energy drinks should be avoided, as they contain large quantities of added sugars and lack nutrients [95]. Energy drinks are also associated with cardiac and psychological issues [95].

Evidence suggests that excessive alcohol intake can dysregulate descending inhibitory pathways and reward network circuitry, which can lead to hyperalgesia [96]. Alcohol also disrupts REM sleep, which can feed into the cyclic relationship between poor sleep and poor eating habits [74,97]. Resveratrol, an antioxidant with anti-inflammatory properties that can be found in red wine may play a role in reducing pain severity [98]. The best advice is to follow national alcohol guidelines such as the National Health and Medical Research Council guidelines in Australia to consume no more than 10 standard drinks per week [99].

### 2.3. Nutrition Considerations for Vulnerable Groups

#### 2.3.1. Older People

Advancing age is major risk factor for developing CNCP. Approximately 20% of adults in the Western world experience CNCP; however, this almost doubles in those aged over 65 years [2,4]. It is also estimated that up to 93% of residents in aged care experience CNCP [100]. As the population ages, the prevalence is expected to increase over time, which will lead to increased healthcare burden and costs.

Malnutrition and dehydration are highly prevalent among older people, especially those in residential aged care facilities. See Table 2 for strategies on how prevent these issues. These nutrition-related issues are also associated with increased risk of experiencing pain [100]. Approximately 50% of older people in Australia are malnourished or at risk of malnutrition, and up to 68% are at risk of dehydration [100]. In addition to an increased risk of experiencing pain, these issues also result in decreased quality of life and increased risk of falls and fractures, sarcopenia, confusion, constipation, and fatigue, all of which further impact the morbidity and mortality of older people [100]. Older people experiencing pain who are malnourished or dehydrated should be referred to a dietitian for medical nutrition therapy [100]. Malnutrition is also associated with deconditioning. This leads to a loss of muscle mass and strength. Consumption of high quality protein combined with resistance and strengthening exercises assist in building muscle mass and strength [101]. In Australia, guidelines state that those aged 70 years and over should consume approximately 1 g protein per kilogram of body weight per day [102]. Consuming high quality protein sources (e.g., lean meat, eggs, nuts, and legumes) across 2–3 meals per day optimises muscle protein synthesis [103].

#### 2.3.2. Culturally and Linguistically Diverse Populations

CNCP disproportionally affects culturally and linguistically diverse populations (CALD), migrants, and refugees [3]. In a cross-sectional study conducted by Kurita 2012 et al., it was reported that the prevalence of CNCP in Danish-born participants was 26%, compared to non-Western born participants in whom the prevalence was 40% [3]. Several studies conducted in Sweden, Switzerland, and Denmark show that immigrants, especially from non-Western backgrounds, have more diagnosed musculoskeletal conditions, higher pain intensity, healthcare utilisation, and increased risk of poor mental health [104]. CALD populations are also less likely to engage in treatment options and have poorer outcomes [104] Systematic reviews frequently limit literature searches to studies published in English and observational and experimental studies often exclude non-English speaking people [104].

There are a variety of complex biopsychosocial and lifestyle factors that influence pain experiences and nutrition practices and beliefs for CALD populations. Some cultures may put different emphases on the relationship between nutrition and the biomedical contribution to pain experiences.

Different cultures have varying beliefs around different foods and their potential role in healing and pain. For example, arthritis may be considered a “hot” condition that needs to be treated with “cooling” foods. In some cultures, food preparation may be a major part of identity, and the loss of ability to express this identity may cause significant distress and worsen an individual’s pain. The impact of language can also directly affect nutrition quality, making it more difficult to shop and read labels, while experiencing pain may also make it more difficult to be able to study and participate in a new culture and language. Culture also affects the types of food eaten, and the manner and volume of eating. In addition, food is frequently an important component of traditions and celebrations.

## 3. Future Directions for Clinical Practice

In order to optimise therapeutic outcomes, pain services should incorporate nutrition screening, assessment, and treatment alongside treatments from other allied health professionals. These should be developed and implemented in conjunction with dietitians and integrated into current pain management practice. Evidence shows that a clinically meaningful reduction in pain can be achieved with personalised dietary advice for patients experiencing CNCP [53]. The whole-person approach to pain management can be strengthened with the inclusion of a person-centred dietary assessment and intervention. Similarly, multidisciplinary teams can also be strengthened with the inclusion of a registered or accredited dietitian to provide this service. It should be acknowledged that while there is dietary advice that can be given to anyone experiencing pain, it is not always a one-size-fits-all approach and dietitians are best placed to provide individualised medical nutrition therapy where needed. In services where this may not be possible, another option is establishing a consultative relationship with a dietitian outside the service. The dietitian can provide their expertise by leading nutrition professional development for clinicians and nutrition programs for individuals and patient groups.

In contemporary practice, dietary assessment of patients attending pain services is uncommon and, therefore, there is very limited information about the dietary intakes and behaviours of patients outside of research studies. To effectively translate research findings from nutrition-based studies, it would be helpful to have a greater understanding of the nutritional status of patients. Collaborations and networks exist to collect data from pain services around the world to assist with benchmarking, but this data does not currently include dietary information.

Exploring the role of telehealth in providing treatments to patients, nutrition-related or otherwise, is also something that should be considered, given some of the barriers patients face in attending face to face appointments [105]. These may include travel time, cost, and accessibility to services. This would extend the reach of dietary treatment to patients who may not currently be able to access it. Contingent upon a viable funding model, nutrition education and behaviour change can easily be delivered via telehealth and there is evidence to support its use for many chronic health conditions [105].

Advocacy needs to continue for the role of nutrition in CNCP management. This can be done at a local, national, or international level through pain services, national pain societies, government prevention strategies and strategic plans, dietetic organisations, consumer groups, and the International Association for the Study of Pain.

## 4. Future Directions for Research

It is evident from the included studies that gaps exist in research that has been undertaken to explore the relationships between nutrition and pain management. The systematic reviews found that the heterogeneity among studies made it difficult to draw strong conclusions. Future intervention studies need to include larger, higher powered sample sizes and test both the efficacy and effectiveness of the intervention. It would also be valuable to include other outcomes such as physical function, psychological measures, biomarkers of inflammation, blood glucose, blood lipids, and blood pressure to determine the effect of interventions on comorbid mental and physical health conditions.

Another limitation of current evidence is the lack of information provided on the intervention components and methods of the included studies. It is therefore difficult to extract precise information on intervention content, mode and frequency of delivery, and the qualifications of the person who delivered the intervention. For example, interventions may be categorised as vegetarian or vegan without specifying critical components such as amounts of sugar and/or refined grains. All of this information is required in order to replicate interventions and translate findings into clinical practice. If future studies better report methodologies and results it would lead to more consistent analysis and synthesis of outcomes and more meaningful interpretation.

The implementation of routine dietary assessment is another important consideration for chronic pain trials. Use of consistent and comprehensive assessment tools is key. Participant burden can be reduced by utilising tools which incorporate technology such as image-based food records. Many of the studies included in the systematics reviews used a unidimensional measure of pain, such as a visual analogue scale. Multidimensional tools that incorporate pain interference and pain self-efficacy provide a more comprehensive measure of pain outcomes, especially in the context of the whole-person approach.

When translating the research into clinical settings, one should consider using co-production or co-design methods to engage and include stakeholders in the development and undertaking of research studies to ensure that the intervention is feasible and acceptable to the local context. Engaging stakeholders will also ensure that the intervention is appropriate and relevant for their needs and wants. With an increased focus on knowledge translation and implementation science, these types of studies are required to ensure that interventions work in the real world.

## 5. Conclusions

Diet should play a pivotal role in pain management. There is a strong link between diet and systemic inflammation and other chronic health conditions associated with CNCP. Best evidence pain management incorporates active strategies that target biopsychosocial and lifestyle factors such as biomedical, mind–body connection, physical activity, sleep, and nutrition. These factors are of variable importance in different individuals and complex inter-relationships exist between them. Nutrition is an area that traditionally has not received sufficient attention in CNCP management. This state-of-the-art paper summarises the relationships between diet, inflammation, comorbidities, and pain management, and uses the current literature to provide recommendations on improving the dietary habits and behaviours of those experiencing CNCP. The paper also proposes future directions for practice and clinical research in this space.

## Figures and Tables

**Table 1:** Best evidence table.

**Experimental Study Designs**	**Level of Evidence [81]**	**Study Type**	**Target Population**	**Intervention(s)**	**Length of Intervention**	**Risk of Bias (ROB)/Methodological Quality**	**Results**	**Evidence Gaps**
Field et al., 2021 [82]	1b	SR and MA (exp); *n* = 43	Chronic non-cancer MSK pain	Veg/vegan (*n* = 11), single food changes (*n* = 11), elimination (*n*-11), energy or macronutrient restriction (*n* = 8), omega-3 (*n* = 5), Mediterranean diet (*n* = 2)	Ave 18 weeks (2 weeks–2 years)	RCT’s and pre-post studies: Good (*n* = 7), fair (*n* = 19), poor (*n* = 11)	N= 23/32 controlled studies included in MA. SMD −0.44; 95% CI: −0.63 to −0.24; *p* < 0.0001; I^2^ = 62% (high heterogeneity)	Poor methodological quality, heterogeneity, most common pain measure unidimensional
Elma et al., 2020 [83]	1b	SR (exp and obs); *n* = 12	Chronic MSK pain	Exp studies: Veg/vegan (*n* = 4), weight loss (*n* = 2), peptide diet (*n* = 1), aspartame elimination (*n* = 1), low FODMAP (*n* = 1)	Exp studies: Ave 16 weeks (4 weeks–1 year)	RCT’s: Good (*n* = 1), fair (*n* = 1), poor (*n* = 3)	7/9 exp studies reported pain relieving effect of dietary changes. Two studies reported no effect (aspartame elimination and vegetarian).	Poor methodological quality, heterogeneity, most common pain measure unidimensional
Genel et al., 2020 [84]	1b	SR and MA (exp); *n* = 7	Arthritis	Mediterranean diet (*n* = 4), anti-inflammatory food (*n* = 2), low inflammatory diet (*n* = 1)	Ave 17 weeks (12–24 weeks)	RCTs: low ROB (*n* = 1), high ROB (*n* = 4), non RCTs: moderate risk (*n* = 1), serious risk (*n* = 1)	Overall no significant change in pain. Subgroup analysis for RA had reduction, SMD −2.81 (95 % CI −3.60, −2.02), *p* <0.00001	Small sample size, poor methodological quality, heterogeneity
Brain et al., 2019 [85]	1b	SR and MA (exp); *n* = 71	CNCP	Altered overall diet (*n* = 16), altered specific nutrient (*n* = 5), supplement-based (*n* = 46), fasting (*n* = 4)	Ave 17 weeks (2 to 2 years)	Positive (*n* =3 1), neutral (*n* = 36), negative (*n* = 4)	MA (*n* = 23): −0.905 (95% CI −0.537 to −1.272), *p* < 0.001 Qual synthesis: 12/16, 2/5, 11/46 and 1/4 studies from each respective group had significant reduction in pain	Poor methodological quality, small sample size, heterogeneity, most common pain measure unidimensional
Silva et al., 2019 [86]	1b	SR (exp); *n* = 7	Fibromyalgia	Weight loss (*n* = 2), vegetarian (*n* = 2), low FODMAP (*n* = 1), gluten free (*n* = 1), MSG and aspartame free diet (*n* = 1)	Ave 18 weeks (4 weeks to 6 months)	All very low or low uncertainty of evidence, except *n* = 1 moderate	All but 2 studies (gluten free and MSG/aspartame free diet) had significant reduction in pain	Poor methodological quality, small sample size
Brain et al., 2019 [53]	1c	Pilot RCT (*n* = 60)	CNCP	Personalised dietary assessment, education (i.e., F&V, good quality fats, antioxidants and micronutrients and fibre) and advice using the Behaviour Change Wheel and provided by a dietitian +/− antioxidant supplement	6 weeks	N/A	All groups had statastically signigicant improvement in pain interference, pain self-efficacy and pain catastrophizing. Personalised dietary support groups had clinically meaningful improvement in pain interference and pain self-efficacy	Small sample size, loss to follow up (30%), placebo effect
Dragan et al., 2020 [87]	2b	Literature Review (exp); *n* = 38	CNCP	Antioxidant, vitamin and minerals (*n* = 9), elimination diet (*n* = 7), energy restriction (*n* = 5), low-fat/plant based (*n* = 5), pre and probiotics (*n* = 5), fruit and fibre (*n* = 4), enriched PUFA (*n* = 2), high protein (*n* = 1)	Ave 15 weeks (4 weeks–1.5 years)	Not reported	Antioxidants, vitamins and minerals: 8 improvement in pain (IP), 1 no difference (ND) Elimination: 4 IP, 3 ND Energy restriction: 5 IP Low fat/plant based: 5 IP Pre/probiotics: 3 IP, 2 ND Fruit/fibre: 4 IP Enriched PUFA: 2 IP High protein: 1 IP (Note IP included a variety of measures e.g., severity or frequency and strength of improvements ranged from trends to significant improvements)	Small sample size, poor methodological quality
Kaushik et al., 2020 [35]	2b	Literature review (*n* = 8)	CNCP	Antioxidant (*n* = 3), Mediterranean diet (*n* = 2), low carbohydrate (*n* = 2), saturated fat (*n* = 1)	Ave 17 weeks (1 day–1 year)	Not reported	Summary of oxidative stress and inflammation provided. Low carbohydrate, 2/3 antioxidant and Mediterranean diet had reduction in oxidative stress and inflammation. 1 antioxidant study showed no change and saturated fat showed increase in oxidative stress and inflammation	Small number of clinical studies, only 2 studies were specifically measured pain, hard to compare dietary studies when variety of interventions
Rondanelli et al., 2018 [88]	2b	Narrative review (*n* = 172)	CNCP	Red wine (*n* = 26), olive oil (*n* = 24), zinc and selenium (*n* = 18), oil seeds (*n* = 14), yoghurt (*n* = 11), F&V (*n* = 10), spices (*n* = 8), vitamin D (*n* = 7), fibre in opioid induced constipation (*n* = 7), cheese (*n* = 7), legumes (*n* = 6), sweets (*n* = 6), omega-3 (*n* = 6), meat and fish (*n* = 5), eggs (*n* = 4), vitamin B12 (*n* = 3), water (*n* = 3), fibre (*n* = 2)	Not reported	Level of evidence: *n* = 1 SR, *n* = 6 RCT or obs study with dramatic effect, *n* = 7 non-RCT, cohort/follow-up studies, *n* = 3 case series, case-control or historically controlled studies, *n* = 149 mechanism based reasoning	A food pyramid was developed and presented as the results of the paper. This divided foods into those that should be consumed daily, consumed 1, 2, or 4 times per week and foods to be eaten occasionally.	Combination of human, in vitro and animal models included, reliance on lower levels of evidence, casual relationships unknown
Towery et al., 2018 [80]	2d	Quasi-exp cohort study (*n* = 14)	Chronic MSK pain	Education on plant based diet and sample menu cycle. Included grains, F&V, legumes, dairy products and eggs. Meat, poultry, seafood and fish not allowed and processed foods and drinks discouraged	8 weeks	N/A	Pain: mean change 3.14 on NPRS (95% CI 2.16–4.12), *p* = 0.0001. Quality of life: mean change 24.991 on SF-36 (95% CI 18.16–31.97), *p* = 0.0001	Small sample (although powered), unable to blind, accuracy of reported intake, convenience sample, self-reported food intake can increase motivation to change eating habits
**Expert Consensus Papers**	**Level of Evidence**	**Study Type**	**Population**	**Summary**				**Evidence Gaps**
Brain et al., 2020 [89]	5b	Expert opinion factsheet	CNCP	Brief summary on how dietary intake effects CNCP (enhancing nervous system and reducing inflammation), reduces or maintains weight, and improves comorbidities Acknowledges how diet can be impacted (and vice versa) by limited mobility and strength affecting shopping, cooking, and preparation of food, mental health issues, feelings of isolation, and lack of sleep Provides tips for nutrition and pain management with a focus on F&V, good quality fats, micronutrient deficiencies, water, fibre, ultra-processed food and added sugar				N/A
Nijs J et al., 2020 [19]	5b	Expert opinion review	CNCP (animal and human studies)	Focus on role of neuroinflammation and the possibility that the interaction between nutrition and central sensitisation is mediated via bidirectional gut-brain interactions Low saturated fat, low added sugar and anti-inflammatory dietary patterns have the following potential therapeutic targets: reduce oxidative stress, preventing toll-like receptor activation, prevent afferent vagal nerve fibres sensing pro-inflammatory mediators, normalise microglial, optimise gut microbiota, reduce polyamine production, and enhance neurotransmitters Beneficial dietary pattern includes polyphenols, fruits, vegetables, and cereals Important focus on long term changes and improvements (pain interference) from dietary changes and does not rely on short term changes (i.e., pain severity)				Need to explore interactions in human studies
Philpot et al., 2019 [79]	5b	Expert opinion editorial	CNCP	Focus on dietary modification to reduce inflammation and therefore alleviate pain. Highlights the potential role of the Dietary Inflammatory Index. Diets high in daily consumption of F&V, olive oil, nuts, and legumes (i.e., Mediterranean-style diet) with adequate micronutrients (omega-3, vitamin B12, and magnesium) in conjunction with a reduction of processed food is anti-inflammatory and potentially beneficial for CNCP Acknowledges challenges faced by patients that impact dietary intake such as financial, physical, and psychological or practice difficulties. Suggests CNCP services would substantially benefit from access to dietitians’ skills in assessment, modification, and support of diets specific to pain patients				Lack of research on the efficacy of diet therapy for people with CNCP and on the barriers to implementing diet therapy into clinical practice.
Bjørklund. 2019 [22]	5b	Expert opinion review	CNCP	Focus on anti-inflammatory compounds (i.e., antioxidants, vitamins, and minerals) and anti-nociceptive/analgesic compounds (e.g., flavonoids and omega-3) Main themes: fruit and vegetables, antioxidants, deficiency of vitamin D, and the ratio of omega-3 to omega-6 Acknowledges additional considerations such as cultural differences, socioeconomic burden, and food availability Despite inconsistency in the literature, diet (in combination with physical activity and a good lifestyle) is still a promising strategy for reducing pain burden and should not be ignored				More research on the best dietary program for CNCP is needed

SR = systematic review, MA = meta-analysis, exp = experimental, obs = observational, MSK = musculoskeletal, RCT = randomised controlled trial, Veg = vegetarian, PUFA = polyunsaturated fats, ROB = risk of bias, MSG = monosodium glutamate, FODMAP = fermentable oligosaccharides, disaccharides, monosaccharides, and polyols, F&V = fruits and vegetables, CNCP = chronic non-cancer pain.

**Table 2:** Nutrition-related tips and strategies to assist older people in managing pain experiences [100].

Monitor Signs for Malnutrition and Risk of Malnutrition	Monitor Signs for Reduced Fluid Intake and Risk of Dehydration	Stimulate Appetite	Increase Fluid Intake	Improve Eating Experience	Reduce Constipation
- Assess and regularly screen older people to determine their nutrition status - Identify changes in weight, food and drink intake and appetite - Identify gastrointestinal symptoms - Monitor changes in mobility and function - Identify psychological disease and/or dementia	- Ongoing pain and dementia may reduce ability and memory - Not feeling thirsty - Inconvenience - Medication side effects - Unable to access drinks - Fluid restrictions	- Offer smaller portions more frequently - throughout the day - Increase fat, protein and/or flavour content - Ensure meals are appealing	- Offer small frequent drinks between meals - Offer foods with higher water content (e.g., soup, fruit, and yoghurt) - Ensure drinks are clearly and easily accessible - Ensure adequate support for drinking and toileting is available if needed - Contraindications: heart failure and fluid restrictions	- Ensure older people have choices at meal times - Find out food preferences and incorporate into meals - Provide eating assistance, where needed - Ensure dining environment is appealing - Do not rush meals	- Encourage high fibre foods, e.g., keep the skin on fruit, high fibre breakfast cereals - Dietary supplements (e.g., psyllium husk) can be added to foods if needed - Laxative and/or stool softening agents may be needed. Increased fluid is also required for these to be effective - Beverages containing sorbitol (e.g., prune or pear juice)

## Data Availability

Not applicable.

## Appendix A. Key Messages and Practical Nutrition Tips for Pain Management

(adapted from Brain et al., 2020 [89])

**Key Messages:**
  1. People with chronic non-cancer pain (CNCP) should be encouraged to consume:
    - A wide range of nutrient-dense foods (e.g., fruits, vegetables, whole grain breads and cereals, meat, dairy, and their alternatives) to ensure they are meeting their nutritional requirements.
    - The recommended amount of fruit and vegetables should be based on your country’s dietary guidelines and to focus on consuming a rainbow of colors every day.
    - Long chain and monounsaturated fats (e.g., omega-3 and olive oil).
    - More fibre and fluid. Adult females should consume 25 g/day of fibre and adult males 30 g/day. Adults should aim for 2–3 L of water per day.
    - Less ultra-processed foods and foods containing added sugars.
  2. Find resources and strategies through your country’s dietetic organisation such as Dietitian Australia, the Academy of Nutrition and Dietetics, The European Federation of the Associations of Dietitians, or the British Dietetics Association that support this information and that you can provide to patients that will assist them.
**Practical Tips for Conducting Dietary Assessments**:
  - Assist patients to screen their diet quality using tools such as the Healthy Eating Quiz
  - If you are concerned about potential nutritional deficiencies consider referring patients to a dietitian for a comprehensive dietary assessment and personalised advice and support.
**Practical Tips to Optimize Diet Quality**
  - Become familiar and learn about your country’s dietary guidelines.
  - Use an inclusive approach, emphasise important foods that should be added (e.g., vegetables), rather than focusing on foods that should be removed (e.g., energy-dense snack foods).
  - Ensure that nutrition-based education is aimed at improving diet quality, as this will address systemic inflammation and enhance pain management.
  - Be aware that this is a broad approach and there may be individualized variation. For personalized dietary advice patients should be referred to a dietitian.
  - Educate patients on the role of vitamins and minerals in pain management and food sources of these nutrients. For example, good dietary-sources of Vitamin D include fish and eggs, good sources of Vitamin B12 are meat, fish, and dairy, and magnesium can be found in green leafy vegetables and whole grains.
  - Encourage patients to spend some time outside to obtain Vitamin D from sun exposure. For most people, 10–15 min of sun on the arms and legs most days of the week will provide most of the Vitamin D required. However, this will vary based on location and the time of year.
**Practical Tips for Fruit and Vegetable Consumption Which are Rich in Phytonutrients to Reduce Oxidative Stress**
  - Educate your patients on the important role of fruits and vegetables in pain management.
  - Encourage your patients to buy in-season fruits and vegetables and to try a new fruit or vegetable each week where possible.
  - If preparation and cooking is an issue for your patients, encourage them to include variety by using frozen mixed vegetables or reduced-salt canned vegetables (e.g., tomatoes and lentils), which can be easily incorporated into meals such as stir-frys, stews, or pasta dishes. Frozen fruits and vegetables are a great option as they maintain their nutritional quality.
  - Work with your patient to come up with ways to incorporate fruit and vegetables into their daily routine, e.g., including vegetables as a snack throughout the day, ensuring half their plate is covered in vegetables at main meals, using frozen berries as a snack, or the addition of yoghurt or cereal.
**Practical Tips for Consuming Long Chain and Monounsaturated Fats (e.g., Omega-3 and Olive Oil)**
  - Educate patients on the role of omega-3 and olive oil in pain management.
  - Communicate, motivate and encourage patients to consume foods high in omega-3 and olive oil.
    ○ Consume oily fish (e.g., salmon and sardines), flax seed oil or canola oil, linseed, and walnuts to boost omega-3 intake. Aim for a minimum of 2–3 servings of oily fish per week.
    ○ Use extra virgin olive oil as the preferred oil in cooking and salad dressings.
    ○ Reduce saturated and trans fats (e.g., butter, processed foods, and hydrogenated vegetable oils).
    ○ Limit polyunsaturated fats high in omega-6 such as sunflower and safflower oils.
  - Supplements: It is preferable to focus on diet quality through food intake rather than via supplements. Seek advice from a dietitian or medical professional if your patient is considering high doses of fish oil supplements. Evidence suggests that 3000 mg of omega-3, over a 3-month period reduces pain experiences, especially in rheumatoid arthritis [106]. There are two types of omega-3 fats in fish oil supplements, EPA and DHA. Supplements which have a ratio of EPA/DHA of ≥1.5 are most beneficial. Suggest good quality brands which contain high doses of omega-3.
**Practical Tips to Increase Fibre and Water Consumption**
  - Encourage patients to consume the recommended serves of fruits and vegetables to increase fibre intake.
  - Provide practical suggestions such as switching to whole meal or whole grain breads, pasta, and breakfast cereals. Keep the skin on fruits and incorporate a variety of mixed vegetables and lentils into meals. Add psyllium husk or bran to meals at breakfast time.
  - Fill a large (1.5 L) drinking bottle with water every day and set a goal to consume it throughout the day.
**Practical Tips to Reduce and Limit Ultra-Processed Food and Added Sugar Intake**
  - Work with patients to swap sugary and energy drinks for water or mineral water flavoured with fresh fruit.
  - Encourage consumption of healthy convenient snacks such as fruit, vegetable sticks, or yoghurt.
  - Incorporate strategies to help patients cook meals at home rather than relying on highly processed conveniently prepared or take away foods.
  - Recommend cooking meals in bulk and freezing the leftovers so patients have a quick, easy, and healthy meal they can have when they have a flare up and do not feel like cooking.
**Practical Nutrition Tips for Vulnerable Populations**
**Older People**
Clinicians and aged care facilities should monitor for signs of malnutrition and dehydration. Assist older people and their families to optimise their food and fluid intake to reduce pain experiences in older people. Some practical tips and strategies can be found in Table 2.
**People from CALD Backgrounds**
  - Be aware of eating patterns and beliefs of the cultural group you are working with (recognising individual variation).
  - Adapt your practice accordingly and include family and community where possible.
  - Culturally informed approaches enhance engagement [107].
